# Utilizing a Population-Genetic Framework to Test for Gene-Environment Interactions between Zebrafish Behavior and Chemical Exposure

**DOI:** 10.3390/toxics10120769

**Published:** 2022-12-09

**Authors:** Preethi Thunga, Lisa Truong, Yvonne Rericha, Jane La Du, Mackenzie Morshead, Robyn L. Tanguay, David M. Reif

**Affiliations:** 1 Bioinformatics Research Center, North Carolina State University, Raleigh, NC 27607, USA; 2 Sinnhuber Aquatic Research Laboratory, Oregon State University, Corvallis, OR 97331, USA

**Keywords:** gene-environment interaction, chemical risk-assessment, zebrafish behavior, toxicity screening

## Abstract

Individuals within genetically diverse populations display broad susceptibility differences upon chemical exposures. Understanding the role of gene-environment interactions (GxE) in differential susceptibility to an expanding exposome is key to protecting public health. However, a chemical’s potential to elicit GxE is often not considered during risk assessment. Previously, we’ve leveraged high-throughput zebrafish (Danio rerio) morphology screening data to reveal patterns of potential GxE effects. Here, using a population genetics framework, we apportioned variation in larval behavior and gene expression in three different PFHxA environments via mixed-effect modeling to assess significance of GxE term. We estimated the intraclass correlation (ICC) between full siblings from different families using one-way random-effects model. We found a significant GxE effect upon PFHxA exposure in larval behavior, and the ICC of behavioral responses in the PFHxA exposed population at the lower concentration was 43.7%, while that of the control population was 14.6%. Considering global gene expression data, a total of 3746 genes showed statistically significant GxE. By showing evidence that heritable genetics are directly affecting gene expression and behavioral susceptibility of individuals to PFHxA exposure, we demonstrate how standing genetic variation in a heterogeneous population such as ours can be leveraged to test for potential GxE.

## 1. Introduction

While chemical exposures have been associated with multiple adverse health effects in heterogeneous populations, individuals within the population may display broad susceptibility differences [1,2]. Such differences in susceptibility are caused by the interplay of genetic and environmental factors, or gene-environment interactions (GxE). Characterizing variability in how individuals from genetically diverse populations respond to chemical stressors is important to protect sensitive groups, especially when faced with an expanding exposome [3]. There are a number of emerging data streams that address this concern of intrinsic variability in response to exposures. For instance, studies carried out using human cell lines obtained from genetically diverse subjects allow us to quantify inter-individual variability in response to drugs and thus help understand the role of gene-environment interactions in eliciting differential susceptibility [4,5]. Risk assessments carried out using in vitro cytotoxicity assays typically apply an “uncertainty” factor of 10 to account for population variation [6,7]. Since this factor is not chemical-specific, and doses are not always relevant to human exposures, making direct connections to human risk can become a challenge. Moreover, the phenotypic repertoire of strictly in vitro approaches does not cover the broad space of potential health effects.

Currently, epidemiological approaches to understand the effect of population genetic variation in health outcomes upon exposure to environmental chemicals have been limited to a few chemicals with high occupational exposures. Even in those few instances, solid estimates of heritable GxE take decades to obtain. There is a need for feasible, cost- and time- efficient methods to assess chemicals’ potential for eliciting differential susceptibility in risk assessment. This is especially important when considering chemicals of emerging public health concern, such as Per- and Polyfluoroalkyl Substance (PFAS).

Toxicity testing using genetically diverse whole-animal models such as zebrafish allows us to explore the extent to which population genetic variation influence toxic responses to chemicals, even for complex traits with small effects sizes [8,9]. Due to their outbred nature, they provide a powerful experimental platform for appropriately accounting for the background genetic variation on which complex disorders manifest. Moreover, their short generation time, high fecundity, and externally developing embryos make them highly useful models for toxicity screening.

Developing zebrafish respond to a wide range of sensory cues early in development. They exhibit spontaneous tail contractions that are sensitive to high-intensity light perturbations by 24 h post fertilization (hpf) in embryogenesis and stereotypical responses to light-versus-dark periods by 120 hpf. Previously, using a large dataset of 1060 ToxCast chemicals, we demonstrated the value of utilizing data from behavioral studies in our genetically heterogeneous T5D population for toxicity assessments [10,11]. Such behavior, modulated by both intrinsic signals and the environment, could be leveraged to flag chemicals having potential to elicit differential population response, but demonstrating heritable genetic effects in a behavioral outcome presents challenges. Foremost, the typical case–control genome-wide association study (GWAS) design used for identifying GxE in binary traits demands large sample sizes and results in high sequencing costs to obtain sufficient power when it comes to detecting GxE in continuous endpoints. This is not practical for the triage stage, where the goal is to confirm putative GxE signals in a subset of chemicals having evidence of potential to elicit susceptibility differences.

In our study, we leveraged a population genetics framework to evaluate how variation in larval behavioral responses and gene expression is apportioned among families exposed to different environments (i.e., control, low dose of PFHxA, and high dose PFHxA). We hypothesized that GxE may be particularly relevant at low doses where the chemical on its own might not be able to induce sufficient toxicity in a measurable outcome but, may significantly contribute to the occurrence of disease in susceptible individuals and thus increase population risk for that chemical. PFHxA is a short-chain member of a sub-group of PFASs called perfluoro carboxylic acids (PFCAs) that is water soluble. Toxicity information on such short-chain molecules is scarce and hence it is still unclear whether these compounds are a safe long-term alternative for the long-chain PFASs in terms of environmental and human health [12]. Here, using a rigorous, family-based study design, we evaluate the differences in intra class correlation (ICC) of larval photomotor response and gene expression between full-sibling families based on different levels of PFHxA exposures. ICC is commonly used as measure of heritability in classical genetic linkage studies [13]. If individuals originating from a single family resemble (i.e., similarity in behavioral response and/or gene expression) each other more than individuals originating from a different family, then the proportion of variance attributed to underlying genetics will be higher in that situation than it would be if all individuals (within and outside) a family responded very differently from each other. Therefore, by independently estimating and then comparing the proportions of phenotypic variance attributed to heritable genetics in different environments, we can assess a chemical’s potential to elicit GxE interactions. We implemented mixed model analysis to test for significance of the gene-environment interaction term on larval behavior and its gene expression upon PFHxA exposure and identified genes that show GxE effect.

## 2. Materials and Methods

### 2.1. Spawning Strategy for the Family-Based Approach

Tropical 5D wild-type zebrafish were housed at Oregon State University’s Sinnhuber Aquatic Research Laboratory (SARL, Corvallis, OR, USA) according to the Institutional Animal Care and Use Committee protocols [14]. Twelve pairs of adult zebrafish were placed into separate tanks and allowed to spawn. Embryos originating from each pair spawn are considered full siblings and referred to as a family. Embryos from each family were transferred to individual wells of 96-well plates. These embryos were developmentally exposed to one of 3 exposure conditions from 6 to 120 hpf to various concentrations of perfluorohexanoic acid (PFHxA; CAS: 307-24-4). PFHxA was purchased from and dissolved using 100% dimethyl sulfoxide (DMSO). The embryos were exposed to either 0.33% DMSO (vehicle control), medium PFHxA (16.4 μM PFHxA), or high PFHxA (74.8 μM PFHxA) with four families per exposure group. The exposures concentrations chosen were based upon results from previous larval behavioral studies carried out by our group and other groups where exposure to PFHxA showed discordant results at the lower concentration [15,16,17]. We hypothesized that population variability could be playing a role in mediating toxic responses, especially in lower concentrations.

### 2.2. Morbidity and Behavioral Assessment

Zebrafish larvae were screened for 2 morphological endpoints at 24 hpf, 10 morphological endpoints 120 hpf, and mortality at both time points. Dead and individuals with abnormal morphology in any of those endpoints were removed from behavioral analysis [18]. At 120 hpf, individuals were subject to the larval photomotor assay (LPR) using Zebrabox behavioral analysis chambers (ViewPoint Behavior Technologies). Larval movements were tracked with motion analysis software for each individual and integrated over 6 s time bins. The assay was carried out for 24 min in total across 4 cycles of 3 min light: 3 min dark. Using custom R scripts, total distance moved in the dark phase was calculated for each individual separately and the statistical techniques used for analyzing this response is described in later sections.

### 2.3. mRNA-Sequencing

**Figure 1:**
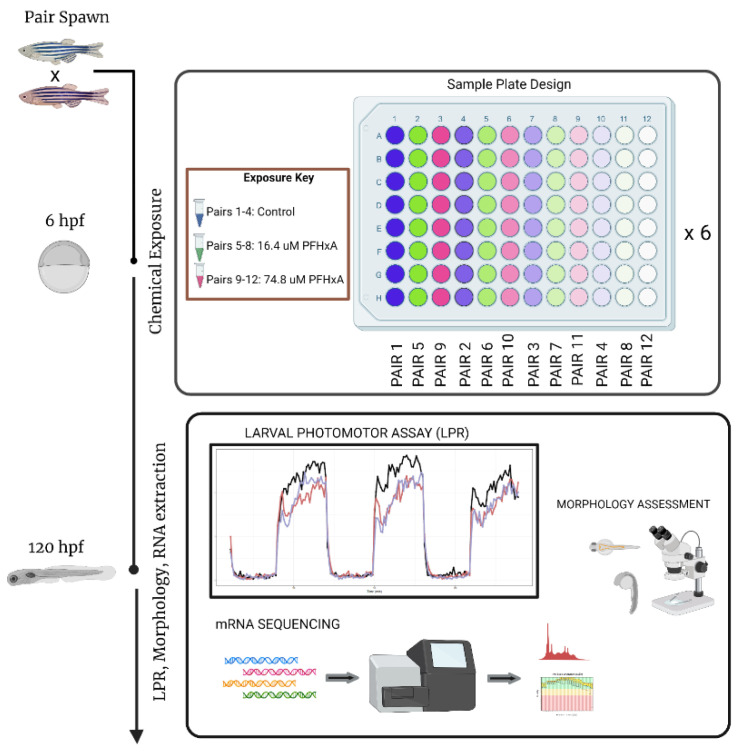
Experimental setup for direct embryonic exposure study (zebrafish embryos exposed to control, 16.4 μM or 74.8 μM of PFHxA from @ 6hpf; phenotypic measurements, i.e., behavioral assessments at 120hpf; developmental assessments of 13 specific morphology endpoints at 120 hpf) for a single test chemical. LPR–Larval Photomotor Response assay. (Figure created with Biorender.com).

To assess potential for gene-environment interaction on gene expression, *n* = 6 larvae per family were randomly chosen, and individually sequenced using Lexogen’s QuantSeq 3′ mRNA-Seq library. A total of 32,171 genes were sequenced in each sample, including 114 spike-in transcripts for quality control. Reads were aligned to Genome Reference Consortium GRCz11 zebrafish reference (https://www.ncbi.nlm.nih.gov/grc/zebrafish) (accessed on 1 February 2022) and genes with low expression levels (gene count < 20) were filtered out. Normalized gene counts were estimated for each gene and modeled as the response term for gene expression analyses. Figure 1 illustrates the timeline of exposure and data collection.

### 2.4. Statistical Analysis

#### 2.4.1. Fitting One-Way Random Effect Model to Estimate ICC

We separated the data by environment (i.e., 4 families per exposure; *n* = 42–28 per family for behavior and *n* = 6 per family for gene expression data) and partitioned variation in different endpoints (behavior and gene expression) using a one-way random effect model. Estimating and comparing the variance estimates among individuals within full sibling families in different environments gives us the intraclass correlation (ICC) statistic, which can be interpreted in a way that is related to heritability. We fit the following simple random effects model for every individual,

Y_ik_ = μ + S_k_ + E_ik_ (1)

where Y_ik_ is the response value (LPR c expression for each gene) of individual i from family k; S is the term accounting for the random variation arising from different families (k = 1, 2, 3, 4) assumed to be N(0, σ^2^_S_) and E_ik_ is the residual error variance. Then, the total variance of an individual, Var(Y_ik_)can be calculated as the sum of the variance attributed to the random effects term and the error variance., i.e.,

Var(Y_ik_) = Var(S_k_) + Var(E_ik_) (2)

σ_T_^2^ = σ_S_^2^ + σ_E_^2^ (3)

We summarized the amount of variance in phenotype attributed to families (i.e., the inter-family variation) as σ^2^_S_/σ^2^_T_ and term this as the intraclass correlation coefficient (ICC). We estimated ICC values for each gene independently in the three environments and used a Wilcoxon-signed rank sum test to test whether the ICC was different between families in different environments. Inter-individual variation within a family can be summarized as σ^2^_E_/σ^2^_T_.

#### 2.4.2. Fitting Linear Mixed Model (LMM) to Assess the Significance of GxE Term

We fit linear mixed models with random slopes and random intercepts independently for different endpoints to model the relationship between the response variable (larval photomotor response or gene expression) and 2 explanatory variables: family ID (genetic) and exposure group (environment). Exposure was modeled as a fixed effect with three levels (control, medium, and high), and family ID was modeled as a random effect. We also included a random interaction term between family ID and exposure to test for the presence of gene-environment interaction. The LMM for each endpoint can be written as:

y_ijk_ = µ + α_j_ + ß_k_ + (αß)_jk_ + ϵ_ijk_ (4)

where, y_ijk_ can be either total distance moved in dark (behavioral endpoint) or normalized gene expression value for a single gene (gene expression data) for an individual i from exposure group j and family k; α = fixed effect due to chemical exposure (j = 1, 2, 3); ß = random effect due to family (k = 1, 2, …, 12) assumed to be N(0, σ^2^_G_); (αß) is the corresponding random interaction effect between family and exposure assumed N(0, σ^2^_GxE_); and ϵ = random error assumed to be N(0, σ^2^_E_) (Table 1).

We used a restricted maximum likelihood (REML) method to fit the model and obtain unbiased estimates of the variance parameters for random effects. To test for gene-environment interaction, for each gene, we determined whether the variance corresponding to the random interaction term in the model (σ^2^_GxE_) is non-zero using parametric bootstrap. The bootstrap involved 1000 resampling based on the true parameter estimates to calculate a 95% confidence interval (CI) for the variance term associated with GxE parameter of interest. A gene was determined to have significant interaction term if the 95% CI excluded zero.

## 3. Results

As a part of pre-processing, larvae showing defects in any of the morphological endpoints were excluded. The number of offspring per family that were retained for modeling of behavior data is shown in Table 2. Distance moved in the first light and dark period was ignored and the remaining data was summed across all light and dark phases independently [10]. We used total distance moved in the dark phase as our behavioral endpoint. As described in Methods, we sampled six larvae from each family total *n* = 72 (6 samples per family, 4 families per treatment, 3 treatments) for mRNA sequencing.

### 3.1. One-Way Random Effect Model to Estimate ICC in Different Environments

**Figure 2:**
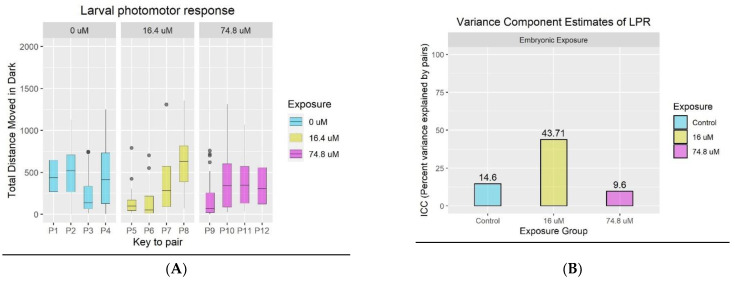
(**A**) Box plot showing total distance moved during dark phase of LPR assay broken down by pairs from each exposure group. (**B**) Variance Component Estimates. Bar plot showing percent of total variance explained by Family ID in each exposure group.

After pre-processing the behavior data, we analyzed data from each exposure group separately. Figure 2A shows the spread of movement values within each family (pair) broken down by exposure. Larvae from pairs 1–4 were exposed to DMSO, those from pairs 5–8 to a medium dose of PFHxA, and offspring from pairs 9–12 were treated with a high dose of PFHxA. This figure indicates that there is a higher amount of variability between different families exposed to medium concentrations of PFHxA when compared to the other two exposure groups. We calculated the intraclass correlation (ICC) for each exposure by fitting a one-way random effects model as described in Section 2.4.1 and these results are shown in Figure 2B. We found that at the medium dose, ICC was 44%, while it was only 15% and 10% in the control and high exposure groups, respectively. We hypothesized that the higher inter-family variance observed in medium dose could be pointing to underlying gene-environment interactions and tested for this using a mixed effects model (See Results Section 3.2).

We then investigated whether this pattern is also observed at the gene expression level using a similar random effect analysis of normalized gene expression counts for each exposure separately. Genes with zero counts across all samples, and genes with counts lesser than 20 in 75% of samples within a given treatment were filtered out. We used two approaches to estimate inter- and intra- family variability: (1) Fitting a large-scale random-effects model to all the gene expression data simultaneously. This was done to apportion sources of variation in gene-expression at an overall level, i.e., to estimate and interpret all the variance components simultaneously; and (2) Fitting a random-effects model for each gene independently and carrying out a Wilcoxon test for differences in percent variance attributed to families in each environment.

The first model is of the form:

y_ijk_ = μ + *β*_1_ · Gene_i_ + *β*_2_ · Family_j_ + Error_ijk_ (5)

Here, gene_i_ is the random effect of gene i (i = 1, 2, …), Family_j_ is the random effect of family/pair j (j = 1, 2, 3, 4) and Error_ijk_ is the residual variance of individual k for gene i belonging to family j. The second model for modeling one gene at a time is simpler as described in Section 2.4.1; response y = family ID + error. The first approach allows us to apportion variance into multiple factors (gene, family, individual) and estimate overall contribution of each term. In the control group, we observed that 91.7% of the variance in response was due to random effect of genes, 8.2% was due to inter-individual variability and less than 0.1% was attributed to families. In the medium group, 90.5% was due to random effect of genes, 9.4% was due to inter-individual variability and less than 0.1% was due to family effect. In the high group, 94.1% was due to random effect of genes, 5.8% was due to inter-individual variability and again less than 0.1% was due to family effect (See Supplementary Tables S1–S3). Due to high variability in expression of different genes, the large proportion of the variances being attributed to random effect of genes is unsurprising. Despite this, we again observed higher levels of inter-individual (residual variance) and inter-family (family ID variance) variability in the medium concentration compared to the other two exposure groups (Supplementary Tables S1–S3).

We used the second approach described earlier to model each gene independently for each treatment group and estimated the proportion of variance in expression that is attributed to family ID. After filtering out genes with low expression levels, roughly 11,000 genes were remaining. Paired Wilcoxon-rank sum test of the variances of these genes within each treatment group showed that the percent variance attributed to family ID was higher in medium exposure groups when compared to the high exposure groups (*p*-value = 0.0016), pointing that especially at lower doses of exposure, genetic variability is likely to increase disease susceptibility. However, the percent variance attributed to family ID for these genes in medium exposure groups was not significantly different from the controls.

### 3.2. LMM to Assess Significance of GxE in LPR and Gene-Expression

LMM analysis of behavioral data estimated variance parameter of the random interaction term in the model to be 0.502 (Table 3). A bootstrapped (*n* = 1000) 95% CI for this parameter was estimated as 0.06–0.728. Because this CI does not include zero, we concluded that there is a significant effect of GxE upon PFHxA exposure in our population.

**Figure 3:**
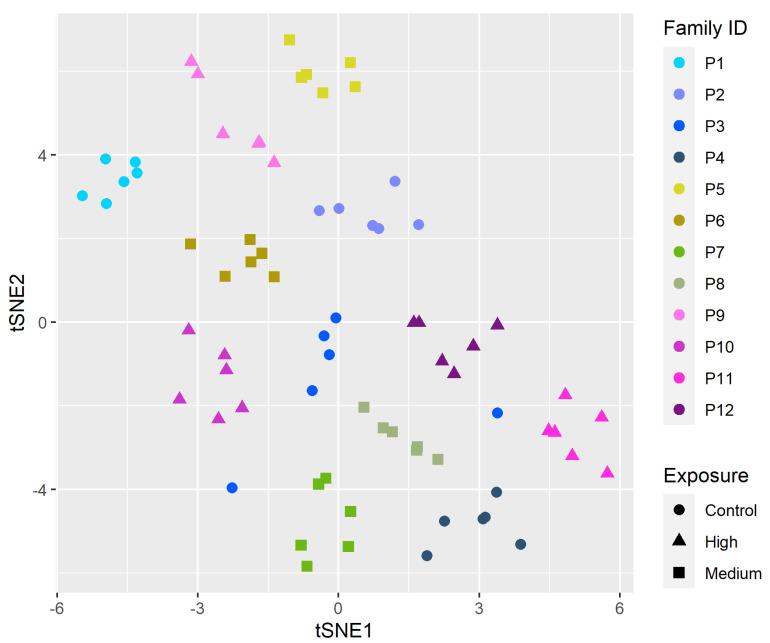
tSNE of normalized gene expression counts of genes that had a significant GxE effect. Color of the points represent family and shape represents exposure. Shades of blue are controls; shades of green represent medium PFHxA groups, and shades of pink represent high PFHxA groups.

Next, we fit LMMs to each gene independently and carried out bootstrapping (*n* = 1000) to assess the significance of the GxE term for each gene. This analysis identified 3746 genes as having statistically significant gene-environment interaction. To visualize how expression of these genes cluster with exposure and family, we carried out tSNE analysis [19] using normalized gene counts of only these 3746 genes (Figure 3). The pattern in Figure 3 shows clusters by family (color) and exposure concentration (shape).

## 4. Discussion

Although it is well-recognized that humans display a wide range of susceptibility differences upon exposure to certain chemicals, potential for a given chemical to elicit gene-environment interactions is rarely specifically quantified during risk assessment. Currently, genome-wide association experiments and epidemiological studies are the two most widely used approaches to characterize effects of genetic variability on disease outcomes. Such studies are limited to only handful of chemicals due to several reasons: high sequencing costs, low effect sizes of most GxE, difficulty to achieve sufficient power, etc. [4,20]. Moreover, most GxE studies carried out in genetically diverse cell-lines are by definition, in vitro, thereby increasing the challenge with regard to extrapolating results to in vivo risk assessments. In our study, we leveraged the population genetic diversity of zebrafish models and designed a family-based exposure study in order to assay the influence of population variability on responses to PFHxA exposure and test for GxE. PFHxA was recently proposed to be listed as a “substance of very high concern” (SVHC) with the European Chemicals Agency (ECHA) due to its high water solubility and potential to contaminate groundwater if left unregulated [21].

We measured and compared shifts in variability of behavioral responses and gene-expression levels between different families within and between environments. Zebrafish behavior, especially at early life stages, is a highly integrative endpoint that is representative of multiple levels of biological organization. Although these assays do not directly point to underlying adverse outcome pathways, aberrant behavioral responses upon exposures to chemicals within this early developmental window indicate that these chemicals are interacting with biological targets to perturb normal development [8,9]. Gene expression serve as a functional link between heritable genetics and observable phenotypes. Taken together, analyzing how variation in these endpoints apportions among and within families allows for the evaluation of how genetic and environmental factors influence responses to chemical exposures. As in Figure 3, samples clearly cluster together based on the families they belong to. In the medium dose of PFHxA, these clusters are “tighter” within families, when compared to families in other exposure groups. In control and high doses of PFHxA, there is more spread within each family. This pattern suggests a GxE effect modifying response to chemical exposure.

There are some limitations to this study. While this analysis provides numerous hypotheses for genes potentially involved in gene-environment interactions upon chemical exposure, it can only suggest putative targets, and therefore further research in a lab setting is necessary to validate their involvement in GxE. Furthermore, future studies could be adjusted to expose individuals from every family to all environments to more clearly separate main effect of family from gene-environment interaction effects. Another limitation is the number of doses tested. While we deliberately selected only two doses—one medium dose that is borderline toxic, and hence could be pointing to a GxE effect, and a high dose that has been shown to significantly alter behavior—we did not carry out an extensive dose–response assessment of variability upon PFHxA exposure. Although the concentrations used in this study are outside those typically considered environmentally relevant for this compound, with the shift in focus of chemical industries toward short chain alternatives such as PFHxA, it is possible that exposure levels may rise in the coming year; and hence future research must probe the ability of similar short chain PFASs to elicit gene-environment interactions.

Pedigree-based studies allow us account for the fact that within a population there maybe differential sensitivity to environmental exposures. A clear benefit of this study is that, on a specific level, we present empirical evidence for an experimental design that could be used to inform estimates of a chemical’s GxE potential. Quantifying the amount of variance in phenotype that is attributable to underlying genetic variability using such an approach during toxicological screens can have a high impact on real-world risk assessment of chemical exposures. In the past, intraclass correlation (ICC) has been used to assess occupational exposure variability and adjust mitigation efforts accordingly [22]. Future work should focus on using similar experimental designs to test for and probe mechanisms of GxE involving compounds of public health interest. This knowledge will aid us to tailor risk management for uniquely sensitive populations.

## 5. Conclusions

Our study used a full-sibling family-based design to estimate relative contributions of heritable genetics to total observed variation in zebrafish behavior and gene expression upon PFHxA exposure. Larval behavioral response and a total of 3746 genes showed significant GxE effects. This illustrates that PFHxA has potential to affect susceptible populations adversely and requires environmental monitoring to ensure exposure levels remain low. We have shown how measuring and accounting for influence of population genetic variability on an individual’s response to chemical exposures could positively impact chemical risk assessment.

## Figures and Tables

**Table 1:** Explanation of parameters fit in LMM.

Parameter	Details
y	Total distance moved (LPR) or normalized gene expression value for a single gene
α	Fixed effect of exposure
ß	Random effect due to family~N(0, σ^2^_G_)
αß	Random interaction term~N(0, σ^2^_GxE_);
ϵ	Residual error~N(0, σ^2^_E_)

**Table 2:** Breakdown of counts of zebrafish larvae remaining from each pair spawn (Family ID). There were 3 exposure groups–4 pairs / exposure, 12 groups in total) after QC.

Family ID	Exposure	Number of Larvae
P1	0.33% DMSO (Control)	45
P2	0.33% DMSO (Control)	42
P3	0.33% DMSO (Control)	45
P4	0.33% DMSO (Control)	48
P5	16.4 μM PFHxA (Medium)	44
P6	16.4 μM PFHxA (Medium)	35
P7	16.4 μM PFHxA (Medium)	48
P8	16.4 μM PFHxA (Medium)	47
P9	74.8 μM PFHxA (High)	42
P10	74.8 μM PFHxA (High)	48
P11	74.8 μM PFHxA (High)	46
P12	74.8 μM PFHxA (High)	48

**Table 3:** Results from fitting LMM to behavioral data.

**Random Effect Variances**		
Group	Variance	Standard Deviation
Family ID	4221 (σ^2^_G_)	64.97
Family ID: Exposure	19,547 (σ^2^_GxE_)	139.81
Residual	77,515 (σ^2^_E_)	278.41
**Random effect parameter estimates**		
Group	Estimate	
Family ID (ß)	0.23	
Family ID: Exposure (αß)	0.50	
Error (ϵ)	278.41	
**Fixed effect parameter estimates**		
Group	Estimate	Standard Error
Intercept	376.54	63.50
Exposure	−0.74	1.43

## Data Availability

The data supporting the conclusions of this article will be made available by the authors upon request.

## Supplementary Materials

The following supporting information can be downloaded at: https://www.mdpi.com/article/10.3390/toxics10120769/s1. Table S1: Results from fitting LMM to Control date; Table S2: Results from fitting LMM to Medium exposure group (16.4 μM PFHxA); Table S3: Results from fitting LMM to High exposure group (74.8 μM PFHxA).
