# Supplementary material for: Utilizing a Population-Genetic Framework to Test for Gene-Environment Interactions between Zebrafish Behavior and Chemical Exposure
Source: Toxics. 2022 Dec 9;10(12):769. doi: 10.3390/toxics10120769 (PMC9781692; doi:10.3390/toxics10120769)
Supplement: Supplementary file 1 [file toxics-10-00769-s001.zip › toxics-1969232-supplementary.pdf]

toxics

MDPI

Supplementary Materials: Utilizing a Population-Genetic Framework to Test for Gene-Environment Interactions between Zebrafish Behavior and Chemical Exposure

Preethi Thunga, Lisa Truong, Yvonne Rericha, Jane La Du, Mackenzie Morshead, Robyn L. Tanguay and David M. Reif

Table S1. Results from fitting LMM to Control data

**Random effect variances ǀ Control group**		
Group	***Variance***	*Standard Deviation*
Genes	1.506e+08 (σ^2_{Gene})	12270.70
Family ID	1.661e+02 (σ^2_{Family})	12.89
Residual	1.348e+07 (σ^2_{E})	3671.80
**Random effect parameter estimates ǀ Control group**		
Group	*Estimate*	
Genes (β_1)	3.34	
Family ID (β_2)	0.0035	
Error (ϵ)	3671.80	
**Fixed effect parameter estimates ǀ Control group**		
Group	*Estimate*	*Standard Error*
Intercept	553.92	84.65

Table S2. Results from fitting LMM to Medium exposure group (16.4 uM PFHxA).

**Random effect variances ǀ Medium group**		
Group	***Variance***	*Standard Deviation*
Genes	1.743e+08 (σ^2_{Gene})	13202.70
Family ID	3.063e+02 (σ^2_{Family})	17.50
Residual	1.809e+07 (σ^2_{E})	4252.80
**Random effect parameter estimates ǀ Medium group**		
Group	*Estimate*	
Genes (β_1)	3.10	
Family ID (β_2)	0.0041	
Error (ϵ)	4252.8	
**Fixed effect parameter estimates ǀ Medium group**		
Group	*Estimate*	*Standard Error*
Intercept	564.44	91.24

Table S3. Results from fitting LMM to High exposure group (74.8 uM PFHxA).

**Random effect variances ǀ High group**		
Group	***Variance***	*Standard Deviation*
Genes	1.849e+08 (σ^2_{Gene})	13596.95
Family ID	2.479e+00 (σ^2_{Family})	1.57
Residual	1.159e+07 (σ^2_{E})	3404.12
**Random effect parameter estimates ǀ High group**		
Group	*Estimate*	

Genes (β_1)	3.99	
Family ID (β_2)	0.00046	
Error (ϵ)	3404.12	
**Fixed effect parameter estimates | High group**		
Group	*Estimate*	*Standard Error*
Intercept	565.09	93.47
